# DNase I targeted degradation of neutrophil extracellular traps to reduce the damage on IgAV rat

**DOI:** 10.1371/journal.pone.0291592

**Published:** 2023-10-31

**Authors:** Xiu-Qi Chen, Li Tu, Qing Tang, Jia-Sen Zou, Xiang Yun, Yuan-Han Qin

**Affiliations:** Department of Pediatrics, The First Affiliated Hospital, Guangxi Medical University, Nanning, China; University of Bari: Universita degli Studi di Bari Aldo Moro, ITALY

## Abstract

**Background:**

In the past two years, studies have found a significant increase in neutrophil extracellular traps (NETs) in patients with IgA vasculitis (IgAV), which is correlated with the severity of the disease. NETs have been reported as an intervention target in inflammatory and autoimmune diseases. This study aimed to investigate the effect of targeted degradation of NETs using DNase I in IgAV rat model.

**Methods:**

Twenty-four Sprague-Dawley rats were randomly divided into three groups: the IgAV model group, the DNase I intervention group and the normal control group, with an average of 8 rats in each group. The model group was established by using Indian ink, ovalbumin, and Freund’s complete adjuvant. In the intervention group, DNase I was injected through tail vein 3 days before the end of established model. The circulating cell free-DNA (cf-DNA) and myeloperoxidase-DNA (MPO-DNA) were analyzed. The presence of NETs in the kidney, gastric antrum and descending duodenum were detected using multiple fluorescences immunohistochemistry and Western blots. Morphological changes of the tissues were observed.

**Results:**

After the intervention of DNase I, there was a significant reduction in cf-DNA and MPO-DNA levels in the intervention group compared to the IgAV model group (all P<0.001). The presence of NETs in renal, gastric, and duodenal tissues of the intervention group exhibited a significant decrease compared to the IgAV model group (P < 0.01). Moreover, the intervention group demonstrated significantly lower levels of renal MPO and citrullinated histone H3 (citH3) protein expression when compared to the IgAV model group (all P < 0.05). The HE staining results of intervention group demonstrated a significant reduction in congestion within glomerular and interstitial capillaries. Moreover, there was a notable improvement in gastric and intestinal mucosa necrosis, congestion and bleeding. Additionally, there was a substantial decrease in inflammatory cells infiltration.

**Conclusion:**

The degradation of NETs can be targeted by DNase I to mitigate tissue damage in IgAV rat models. Targeted regulation of NETs holds potential as a therapeutic approach for IgAV.

## 1. Background

IgA vasculitis (IgAV), formerly known as Henoch-Schönlein purpura, is the most common systemic small vasculitis in childhood involving capillaries, venules and arterioles. It is characterized by the deposition of IgA, complement C3 and fibrin in the blood vessel wall [1, 2]. The presentation of IgAV is characterized by non-thrombocytopenic purpura involving the skin, digestive tract, joints, and kidneys. The incidence rate of IgAV in children is 10-27/100,000 annually [3, 4]. Although IgAV is typically a self-limiting disease, a few cases may experience gastrointestinal bleeding and renal involvement leading to poor outcomes [5].

In the past two years, study have revealed a significant increase in neutrophil extracellular traps (NETs) within the skin of patients with IgAV, which has been found to be closely associated with disease severity [6]. Our previous study [7] revealed the presence of NETs in peripheral blood, kidney, stomach, and duodenum of children with IgAV. NETs can directly damage the tissue or activate target immune cells to produce a plethora of inflammatory factors such as tumor necrosis factor α (TNF-α), interleukin 8 (IL-8), leading to neutrophil aggregation and vascular/tissue damage [8–10]. Additionally, MPO-DNA levels were also significantly elevated in the circulation of IgAV patients and positively correlated with IgA levels, indicating that NETs play a crucial role in the pathogenesis of IgAV [11].

In recent years, NETs have emerged as potential therapeutic targets in inflammatory diseases, autoimmune diseases, and covid-19 diseases [12–15]. It is hypothesized that targeted degradation of NETs could mitigate tissue damage. Therefore, inhibiting the formation or degrading components of NETs may offer a promising approach for treating Ig AV [15]. The aim of this study was investigated whether DNase I can effectively degrade NETs and protect against tissue damage in IgAV rat.

## 2. Methods

### 2.1 Animal, experimental methods and targeted interventions for DNase I

Twenty-four Sprague-Dawley (SD) rats aged 4 weeks and weighting between 100-120g were procured from the Animal Experiment Center of Guangxi Medical University. The rats were randomly assigned to three groups: IgAV model group, DNase I intervention group and normal control group with 8 rats in each group. The IgAV rat model was established according to the previous study [16]. The rats in the IgAV model group were administered a weekly intravenous injection of 40mg/kg Indian ink (Zurui Biotechnology Co., LTD Shanghai, China) via the tail vein once for three weeks. Subsequently, they received a weekly intraperitoneal injection of 1 mL emulsion solution containing 10mg/kg ovalbumin (Sigma) mixed with Freund’s complete adjuvant (Sigma, volume ratio, 1:1) for 6 weeks. At the end of the 9th weeks, simultaneous intravenous injections of 0.2 mL ovalbumin saline (10mg/mL) were administered daily for 3 days in the IgAV model group, while in the DNase I intervention group, simultaneous intravenous injections of 0.2 mg/kg DNase I (3000 U/mg Roche) and 0.2 mL ovalbumin saline (10 mg/mL) were given daily for 3 days. In the normal control group, an equal volume of normal saline was administered at each stage using the same route as the IgAV model group. At the end of the experiment, blood sample were obtained from the abdominal celiac artery following anesthesia. Subsequently, all the rats were euthanized and renal, duodenal and gastric antral tissues were fixed in a 10% formalin solution for subsequent morphological and immunohistochemical analysis.

The experiments were approved by the Ethics Committee of the First Affiliated Hospital of Guangxi Medical University and conducted in accordance with the guidelines of animal welfare of the World Organization for Animal Health.

### 2.2 Immunoglobulins of IgA, IgE, IgG, C3

Blood samples from all the rats were collected using serum tubes for immunoglobulins. The levels of serum IgA, IgG, IgE, and C_3_ levels were detected by using enzyme linked immunosorbent assay (ELISA) according to the manufacturer’s instructions respectively.

### 2.3 Hematoxylin and eosin (H&E) and periodic Periodic Acid-Schiff (PAS) staining

Tissue samples were taken from formalin-fixed, paraffin-embedded blocks for histology and immunofluorescence. For general morphology and immuno-histochemical analysis, sections with a thickness of 5–7μm were stained using hematoxylin and eosin (H&E) as well as PAS staining.

### 2.4 Quantification of circulating cf-DNA and MPO-DNA levels

The cf-DNA was quantified using ds-DNA Quant-iT PicoGreen quantification kit (Invitrogen) and MPO-DNA levels were determined using the MPO-DNA Quantikine ELISA Kit (Cusabio) in according with the manufacturer’s instructions, as described in our previous study [7].

### 2.5 Multiple immunofluorescences staining of IgA, C3 and NETs

The paraffin sections were deparaffinized in xylene for 15 min and dehydrated in a gradient of ethanol (85% and 75% respectively) for 5 min. Subsequently, the sections were incubated at 60–70°C for 15min in ethylene diamine tetraacetic acid (EDTA) antigen retrieval buffer with a pH of 8.0, followed by three washes with phosphate buffer saline (PBS) at a pH of 7.4. To inhibit endogenous peroxidase activity, slides were immersed in 3% H_2_O_2_ and incubated at room temperature for 15 min and then washed three times with PBS. After being coated with 3% bovine serum albumin (BSA) at room temperature for 30 min, the first primary antibodies (Anti-IgA antibody, Absin, Shanghai, China and anti-C3 polyclonal antibody, Absin, Shanghai, China) were added and incubated overnight at 4°C. Subsequently, they were treated with secondary antibodies conjugated to horse radish peroxidase (HRP) at room temperature for 50 min in darkness. The sections were incubated with fluorochrome [CY3-TSA (Servicebio, Wuhan, China), FITC-TSA (Servicebio)] in PBS and kept at 4°C for 50 minutes. At last, all the sections were incubated with DAPI solution at room temperature for 10 min and then exposed to a spontaneous fluorescence quenching reagent for 5 min.

The multiple immunofluorescences staining of NETs and acquisition of fluorescent images from tissue slices were conducted, followed by analysis based on our previous study [7]. Image analysis was performed using the Image J software version 1.51a (NIH, Bethesda, MD). The percentage area occupied by NETs in the tissue was calculated as follows: the area of NETs determined by cit-H3 co-localization with MPO/NE within the tissue per high magnification 40× field.

### 2.6 Western blot detects the expression of citH3 and MPO proteins

The kidney’s total protein was extracted using the cytonuclein/plasma protein extraction kit (Sangon Biotech, Shanghai China). The protein concentration was determined using the BCA kit (Thermo Fisher Scientific). Protein isolation was performed using 5% and 12% sodium dodecyl sulfate polyacrylamide gel electrophoresis (SDS-PAGE). Subsequently, the protein was transferred to the polyvinylidene fluoride (PVDF) membrane through the wet transfer method. The PVDF membrane was blocked by soaking it in 1% BSA blocking solution for 2h, followed by incubation with primary antibody cit-H3 (Abcam) at a dilution of 1:1000 and MPO (Abcam) at a dilution of 1:1000. After being incubated overnight at 4°C and washed with PBS 3 times, the horseradish peroxidase labeled secondary antibody (1:5000, Thermo Fisher Scientific) was added and incubated at room temperature in dark for 1.5 hours. PVDF membrane was scanned using the FluorChemHD imaging system. The ratio of the gray value of the target protein band to the gray value of the GAPDH band was analysised by using AlphaView.

### 2.7 Statistical methods

Statistical analysis was performed using the Statistical Package for the Social Sciences software, release 19.0 for Windows (SPSS 19.0, USA). Normality distribution of data was assessed by Shapiro-Wilk test. Measurement data were presented as $\bar{X}$ ± SD. The statistical analysis of the quantitative data was analyzed using one-way ANOVA between more than two groups and followed by a SNK test. A *P* value of <0.05 indicated statistical significance.

## 3. Results

### 3.1 Immunoglobulins changes in IgAV

**Fig 1:**
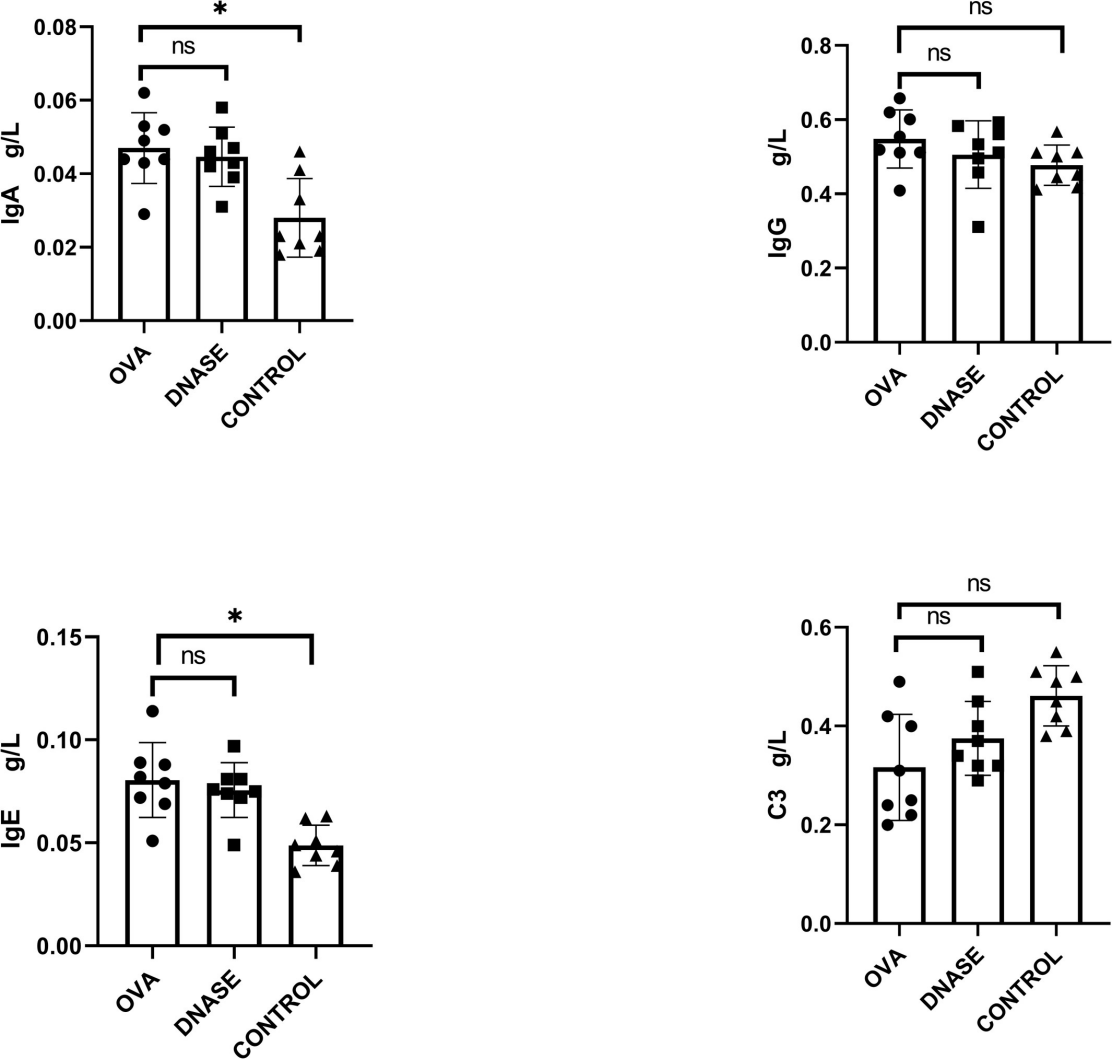
Comparison of immunoglobulins and C3 in IgAV model group, DNase I intervention group and control group. The data showed that IgE and IgA in IgAV model group were significant elevated compared with the control group. After DNase I intervention, IgE and IgA were showed no change between the IgAV model group and DNase I intervention group. The C3 in IgAV model group were significantly reduced compared with the control group. After DNase I intervention, C3 was showed no change between the IgAV model group and DNase I intervention group. DNase, DNase I intervention group. OVA, IgAV model group. * P<0.05, ns P>0.05.

The levels of IgA and IgE were significant elevated in both IgAV model group and DNase I intervention group compared to the control group (*P* < 0.05). Additionally, the C3 levels was significantly reduced in both IgAV model group and DNase I intervention group compared to the control group (*P* < 0.05). However, there was no significant difference observed in the levels of IgA, IgE and C3 between the IgAV model group and DNase I intervention group, *P* > 0.05(Fig 1).

### 3.2 Changes in cf-DNA after DNase I intervention

**Fig 2:**
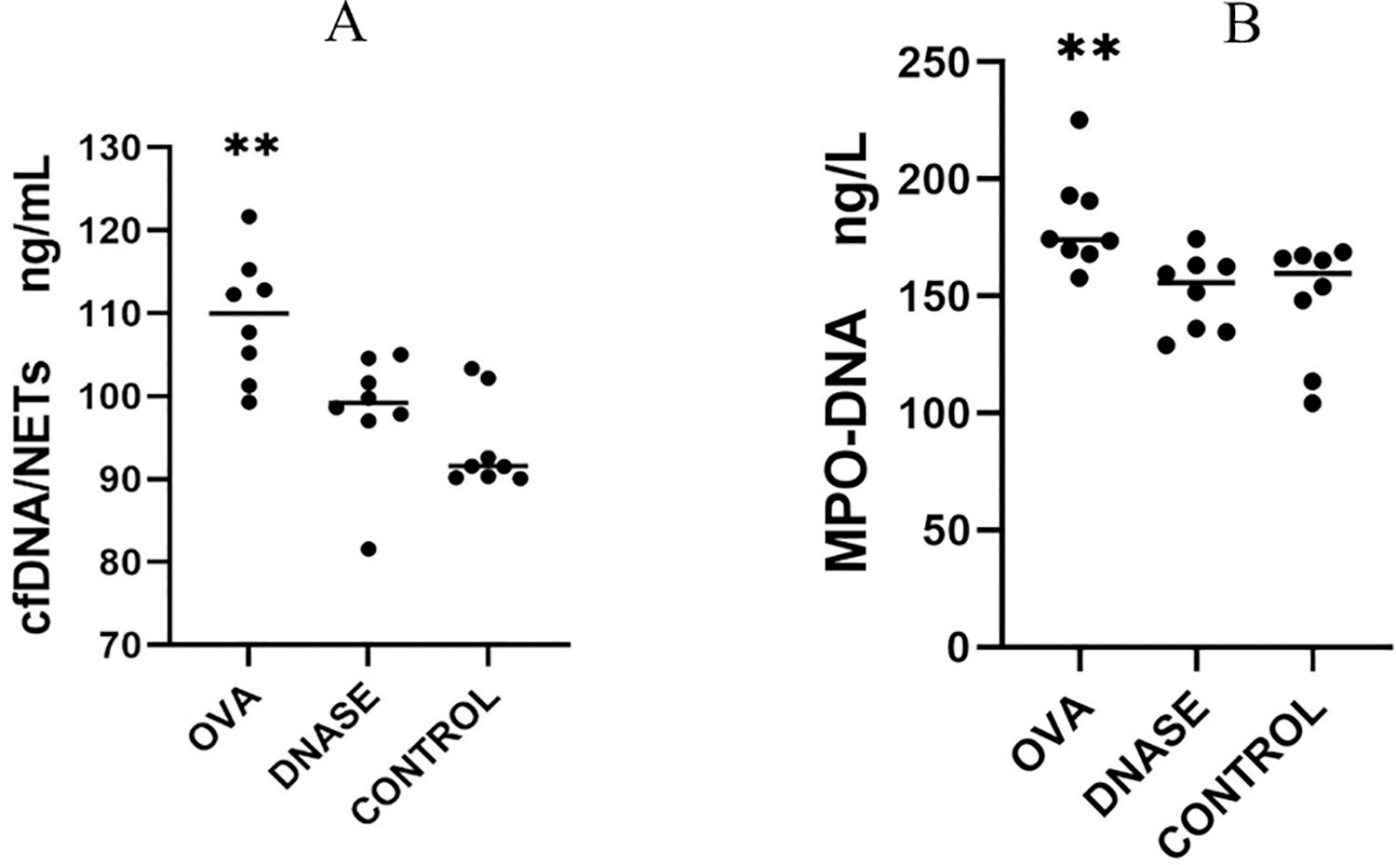
A, Comparison of cf-DNA. B, Comparison of MPO-DNA. The data showed that cf-DNA and MPO-DNA in IgAV model group were significant elevated compared with the control group. After Dnase I intervention, cf-DNA and MPO-DNA were significant decline in the DNase I intervention group compared to the IgAV model group. **P<0.05 compared to intervention group and control group. DNase, DNase I intervention group. OVA, IgAV model group.

The concentration of cf-DNA in peripheral blood was 109.44 ± 7.45 ng/mL in the IgAV model group, 98.26 ± 7.36 ng/mL in the DNase I intervention group, and 93.97 ± 4.49 ng/mL in the control group. One-way ANOVA analysis revealed a significantly difference in cf-DNA levels among the three groups, *P*<0.001. The cf-DNA was as significantly lower in the DNase I intervention group compared to the IgAV model group, *P*<0.0001. there was no statistically significant difference in cf-DNA between the control group and DNase I intervention group, *P* > 0.05 (Fig 2A).

### 3.3 Changes in MPO-DNA after DNase I intervention

The MPO-DNA concentration was 181.62 ± 21.1 ng/L in the IgAV model group, 151.44 ± 16.39 ng/L in the DNase I intervention group, and 148.48 ± 25.52 ng/L in the control group.

One-way ANOVA analysis showed a significant difference in MPO-DNA levels among the three groups, (*P* = 0.009). The MPO-DNA concentration was significantly lower in the DNase I intervention group compared to the IgAV model group (*P* < 0.001). There was no significant difference in MPO-DNA levels between the control group and the DNase I intervention group (*P* > 0.05) (Fig 2B).

### 3.4 Protein levels of MPO and citH3

**Fig 3:**
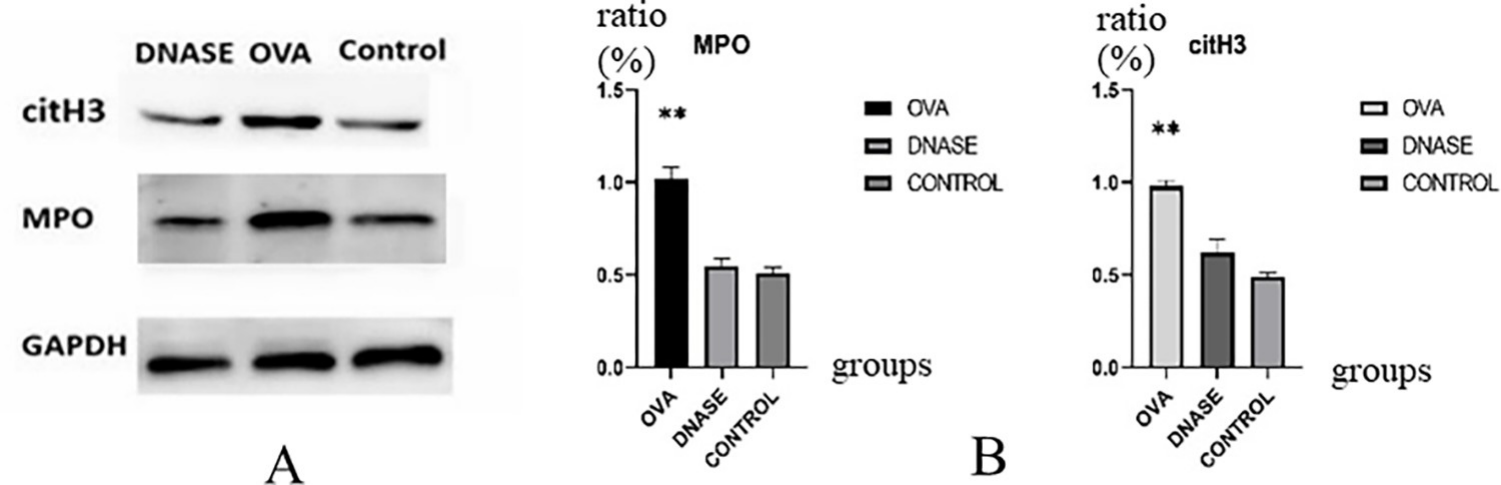
The protein expression of MPO and citH3 in renal tissues. A, Western blot electrophoresis band of MPO and citH3 protein. B, Quantitative analysis of MPO and citH3 protein. ** P<0.01 DNase, DNase I intervention group. OVA, IgAV model group. After Dnase I intervention, the MPO and citH3 were significant decline in the DNase I intervention group compared to the IgAV model group. The MPO and citH3 were showed no significant change between the DNase I intervention group and control group.

The expression of citH3 and MPO proteins in renal tissue was significantly increased in the model group. After DNase I intervention, the levels of citH3 and MPO protein expression in the DNase I intervention group were restored to normal level (Fig 3A). Compared to the IgAV model group, there was a significant decreased in citH3 and MPO protein expression in the DNase I intervention group, with all *P* < 0.05 (Fig 3B). There were no significant differences between the control group and the DNase I intervention group, with all *P* > 0.05 (Fig 3B).

### 3.5 Changes in the expression of NETs

**Fig 4:**
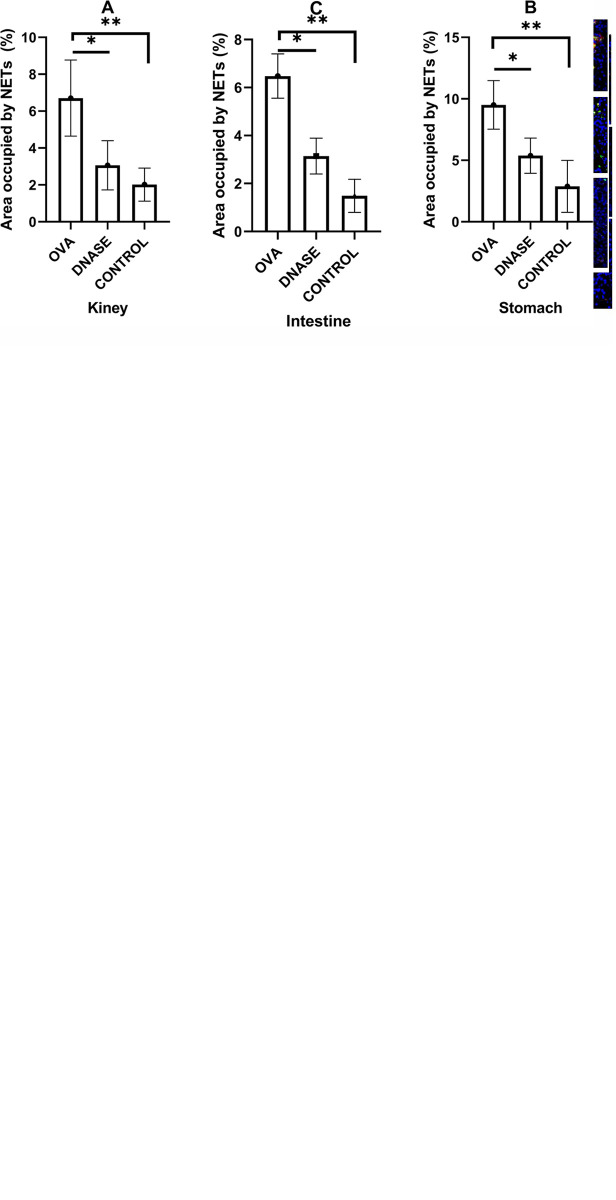
a. NETs in kidney tissues. b. NETs in intestine tissues. c. NETs in gastric tissues. Scale label 50 μm. NETs were observed in IgAV model group and decreased in DNase I intervention group but absent in control group. The sections were labeled for extracellular DNA, and cit-H3 was seen to co-localize with MPO/NE in the mesangial area of the glomeruli, gastric mucosa and submucosa tissues, and duodenal mucosa and submucosa tissues. (green: MPO, red: NE, pink: citH3). d. Area occupied by NETs in renal Kidney, descending part of duodenum and gastric antrum, as determined by image analysis. **P < 0.01, *P < 0.05. OVA, IgAV model group. DNase, DNase I intervention group.

The model group exhibited the presence of NETs in renal, gastric and duodenal tissues as demonstrated by multiple fluorescence immunohistochemistry results. After DNase I intervention, the presence of NETs in the DNase I intervention group was significantly reduced compared to the IgAV model group (Fig 4A–4C). The area occupied by NETs within the positive stained region in all the tissue was significantly decreased in DNase I intervention group compared to the control group, with all *P* < 0.05 (Fig 4D). In contrast, no NETs were detected in the control group.

### 3.6 Histopathological changes in IgAV

**Fig 5:**
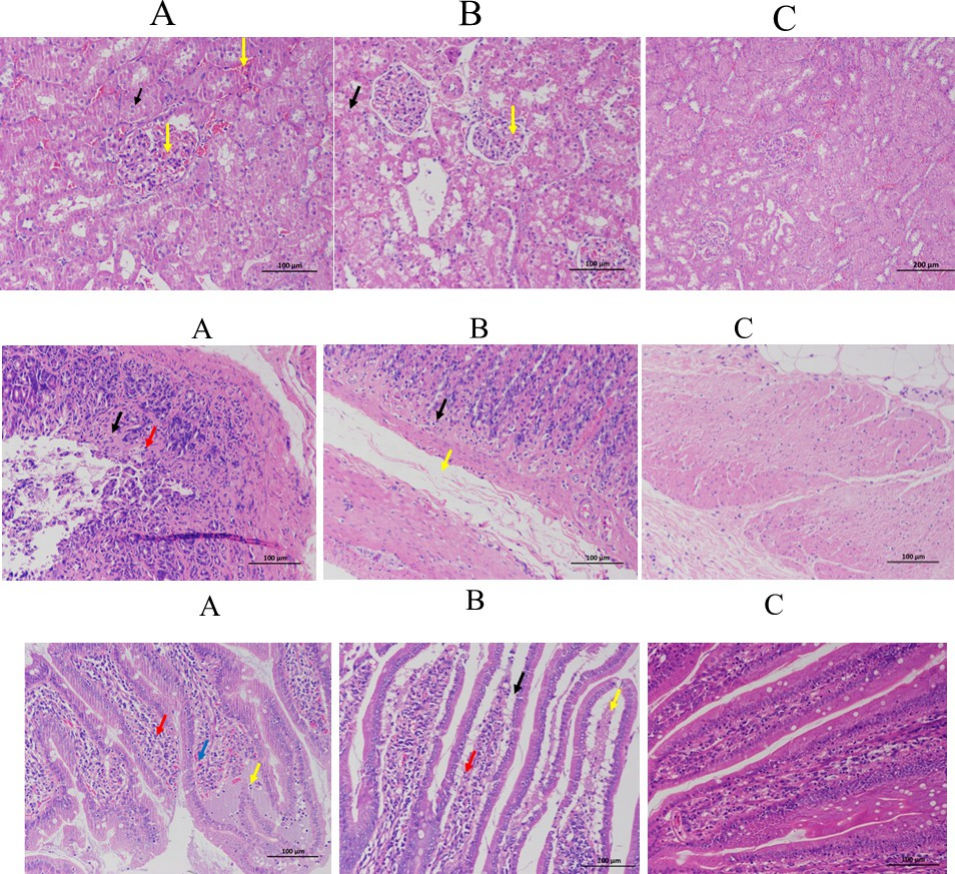
a: Pathological changes of renal tissues. (HE staining, 200×). scale label 50 μm. A, IgAV model group: A large number of renal tubular epithelial cells with watery degeneration and loose cytoplasm (black arrow). There was a large amount of capillary congestion in the glomeruli (yellow arrow). B, DNase I intervention group: The above description was significantly reduced. C, Control group: Normal histology tissue. b: Pathological changes of gastric tissues (HE staining, 200×). A, IgAV model group: Focal necrosis was observed in the mucosal layer of gastric tissue, necrosis of gastric glands disappeared, accompanied by inflammatory cell infiltration (red arrow), infiltration of eosinophils at the bottom of lamina proper (orange arrow), necrotic dissolution of smooth muscle of mucosal muscle (blue arrow). Submucosal edema and loose connective tissue arrangement (yellow arrows). B, DNase I intervention group: the above description was significantly improved. C, Control group, normal histology tissue. c: Pathological changes of intestinal tissues (HE staining, 200×). A, IgAV model group, A large number of intestinal villous epithelial cells were necrotic and shed in the mucosal layer of intestinal tissue, and the lamina propria was exposed (black arrow). In the lamina propria, capillary extravasation was observed (yellow arrow). A small number of intestinal glands were necrotic and disappeared (blue arrow), accompanied by inflammatory cell infiltration (red arrow). B, DNase, DNase I intervention group: the above description was significantly reduced. C, Control group: basically normal.

**Fig 6:**
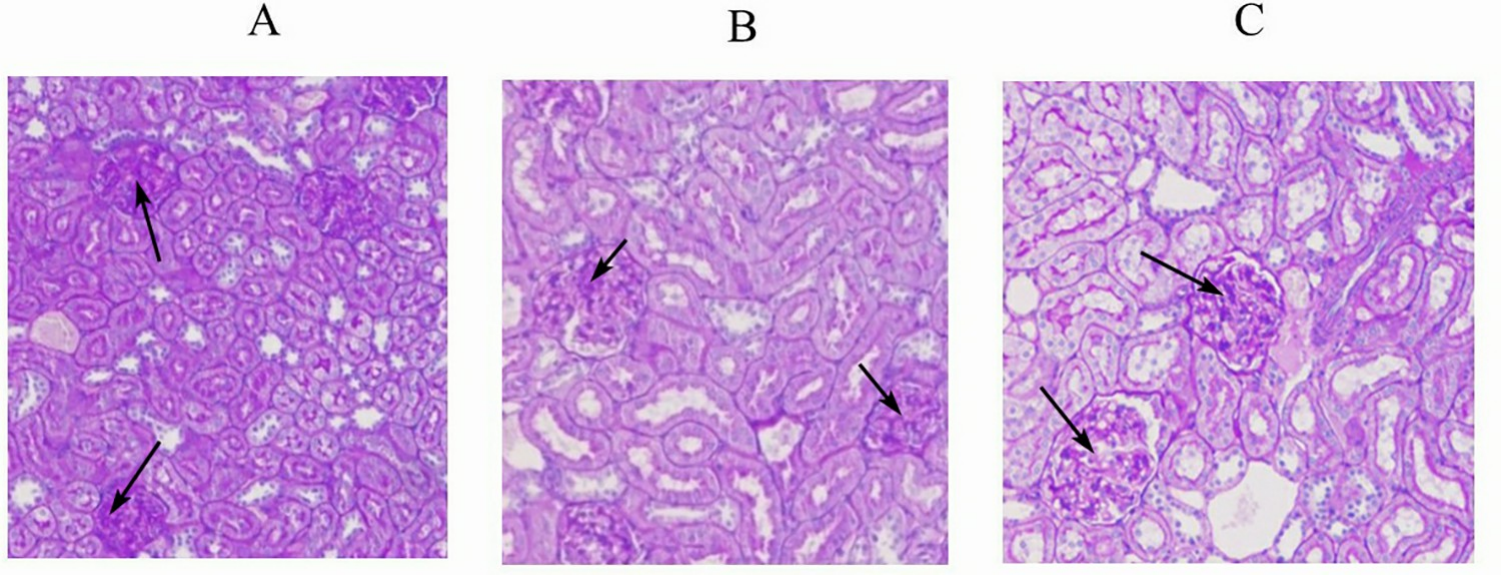
Renal PAS staining results (200×) A, IgAV model group. B, DNase, DNase I intervention group. C, Control group. Compared with the model group, the mesangial thickening, mesangial cell and matrix hyperplasia were significantly reduced in the intervention group.

In the IgAV model group, discrete foci of necrosis were scattered throughout the renal tissue with congestion observed in both stromal and glomerular capillaries (Fig 5A). PAS staining revealed mesangial thickening, mesangial cell and stromal hyperplasia, cystic protein exudation, and focal glomerulonephritis in the model group (Fig 6). All these changes were significantly alleviated after DNase I intervention. Mucosal hemorrhage, epithelial cell shedding and necrosis, as well as inflammatory cells infiltration were detected in the gastric tissue (Fig 5B). Epithelial cell shedding and necrosis, villous dilation congestion, and bleeding accompanied by inflammatory cell infiltration were found on duodenal tissue (Fig 5C). These alterations in both gastric and duodenal tissue were significantly relieved after DNase I intervention.

## 4. Discussion

Studies have demonstrated the direct targeting effect of DNase I on the degradation of NETs [17, 18]. DNase I has been utilized in vivo to degrade the DNA skeleton and treat systemic lupus erythematosus (SLE) and cystic fibrosis [19, 20]. As a component of the host defense system, DNase I can effectively degrade the DNA skeleton within NETs, making it a potential therapeutic target for NETs [15, 21]. The present study directly employed DNase I to degrade cf-DNA, the backbone component of NETs in IgAV rat. After intervention with DNase I, there was a significant decrease observed in cf-DNA and MPO-DNA levels, indicating that DNase I has the ability to directly degrade components of NETs in IgAV. Our previous study confirmed that DNase I levels was significantly reduced in children with IgAV, which may result in an inability to degrade NETs and lead to tissue damage [7]. Meanwhile, the serum of IgAV patients could not effectively degrade NETs. The results were similar to other autoimmune diseases such as SLE, ANCA-associated and large vessel vasculitis which also have a significantly lower degradation capacity of NETs and elevated levels of NETs during active disease [22–25]. The presence of DNase I inhibitors or anti-NETs antibodies is speculated to impede the degradation of NETs, potentially contributing to their persistence. The reduced activity of DNase I and the dysregulation in NETs degradation have been associated with the severity of lupus nephritis [22]. The study revealed that pediatric COVID-19 with impaired NET degradation exhibited elevated levels of G-actin, a natural inhibitor of DNase1, as well as anti-NET antibodies. Serum samples from multisystem inflammatory syndrome displayed reduced ability to degrade NETs, which was association with the presence of G-actin or anti-NET antibodies, respectively, but not with genetic variants of DNases [26].

This present study demonstrated significant decrease in the expression of NETs in renal and gastrointestinal tissues after intervention with DNase I. Pathological findings also showed a notable reduction in tissue damage after DNase I intervention. The results indicate that DNase I can effectively degrade NETs in IgAV, thereby mitigating its detrimental effects. Furthermore, there is no evidence suggesting an association between decreased activity of DNase I and gene polymorphisms [26, 27]. Importantly, It has been confirmed that DNase I directly degrade NETs to prevent the occurrence of vascular thrombotic occlusion [28]. In a mouse model of acute endometritis, DNase I has been shown to inhibit the formation of NETs and reduce TNF-α levels through the NF-κB signaling pathway [29]. In an SLE mouse model, recombinant DNase I significantly improved renal histology and prolonged the survival time by degrading the DNA skeleton [30]. Additionally, DNase I can decrease B cell activity, IFN production, and immune complex deposition in the kidneys [31]. Our study findings align with the therapeutic effects observed in SLE mice treated with DNase I. However, another study have reported that administration of DNase I does not extend survival time in SLE mice [32].

The findings of this study suggest that DNase I has the capability to directly degrade NETs in Ig AV rats, leading to significant reduction in the level of NETs and tissue damage. Therefore, targeting NETs could be a potential therapeutic approach for intervening in the treatment of IgAV. Currently, glucocorticoids and immunosuppressants remain the mainstay for managing refractory IgAVN. However, recent research has focused on exploring biological agents such as monoclonal antibody rituximab for treating IgAVN [33–36]. Rituximab primarily targets CD20 on B lymphocyte, and its mechanism in the treatment of IgAV remains unknown. In addition to direct degradation of cf-DNA in NETs by DNase I, inhibitors of peptidyl arginine deiminase 4 can also directly break down the histones, where aer components of NETs [37, 38]. Furthermore, eculizumab can inhibit the formation of NETs through complement C5 pathway. Vitamin D can also directly reduce the formation of NETs. These represent potential targets for degradation of NETs [15, 39, 40]. However, further research is needed to explore targeted degradation of NETs in the treatment of IgAV.

Besides the DNase1, DNase1-like 3 also degrades NETs in circulation and tissues. In the absence of both DNases, intravascular NETs formed clots that obstructed blood vessels and caused organ damage. Extracellular degradation of NETs by DNase1L3-mediated has been observed in Dendritic Cells [41]. Study have also showed independently expressed of both DNase1 and DNase1-like 3, providing dual host protection against the detrimental effects of intravascular NETs [26, 28].

Previous views have held that DNase I exhibits poor stability in serum and the inability to monitor its activity in vivo limits its clinical application [42]. However, recent study has shown that conjugating DNase I to microgels synthesized from highly hydrophilic N-(2-hydroxypropyl) methacrylamide (HPMA) and zwitterionic carboxybetaine methacrylamide (CBMAA) (DNase I-MG) enhances both the biological activity and stability of DNase I.

This novel approach, utilizing DNase I-MG nanoparticles on microgels, enables a more stable and efficient degradation of NETs compared to free DNase I, significantly improving their degradation efficiency [43]. Another study proposed a novel strategy to enhance the eradication of bacterial biofilms by utilizing large pore mesoporous silica (MSN) nanoparticles doped with Ag nanoparticles (MSN-Ag-DNase I) for the delivery of DNase I [44]. Therefore, the synthesis of DNase I nanoparticles through biological hybridization offers promising prospects for targeting NETs degradation in the clinical disease’s treatment.

In conclusion, the degradation of NETs can be targeted by DNase I to mitigate tissue damage in IgAV rat models. Targeted regulation of NETs may be potential therapeutic method for refractory or recurrent IgAV.

## Supporting information

S1 File (RAR)
